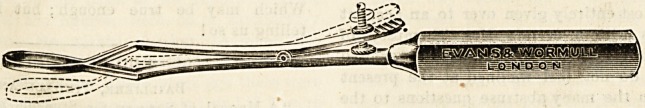# New Appliances and Things Medical

**Published:** 1902-11-01

**Authors:** 


### NEW APPLIANCES AND THINGS MEDICAL.

### COMBINED SHARP AND BLUNT UTERINE CURETTE.

(Messrs. Evans and Wormull, Stamford Street, S.E.)

This double curette has been made after the design of Mr. Alexander Dutar. The curette is of the usual shape, but outside the inner or blunt curette another one, with sharp cutting edge is accurately adjusted. If after the curette has been passed into the uterus it is thought necessary to employ a cutting instrument, by means of a milled nut the sharp curette has been released from its sheath and regulated to a nicety. In passing the curette the blunt edge of the inner blunt blade should project beyond the outer or cutting blade. Any danger of lacerating the cervix is thus avoided.

### MONTREUX MINERAL WATER.

(Health Resorts Bureau, 27 Shoe Lane, E.C.)

This natural alkaline water contains representatives of the earthy carbonates, as well as the carbonates of potassium and sodium. It contains also small quantities of sulphate of calcium and traces of various other salts. It is highly charged with carbonic acid. Montreau water, though essentially a table water, possesses mild medicinal effects, stimulating the action of the stomach, aiding digestion, promoting peristalsis, and relieving dyspepsia. It is a very pleasant and safe water for mild cases of gout and rheumatism.

## Figures and Tables

**Figure f1:**